# Experimental Investigation of Durability Properties of Polymer Coated Pumice Aggregate Lightweight Concretes

**DOI:** 10.3390/polym17020253

**Published:** 2025-01-20

**Authors:** Metin Tuncer, Alper Bideci, Bekir Çomak, Gökhan Durmuş, Özlem Sallı Bideci

**Affiliations:** 1 Department of Civil Engineering, Faculty of Engineering and Natural Sciences, Antalya Bilim University, 07190 Antalya, Türkiye; metin.tuncer@antalya.edu.tr; 2 Department of Architecture, Art, Design and Architecture Faculty, Düzce University, 81620 Düzce, Türkiye; ozlembideci@duzce.edu.tr; 3 Department of Civil Engineering, Engineering Faculty, Düzce University, 81620 Düzce, Türkiye; bekircomak@duzce.edu.tr; 4 Department of Civil Engineering, Faculty of Technology, Gazi University, 06560 Yenimahalle, Türkiye; gdurmus@gazi.edu.tr

**Keywords:** lightweight concrete, pumice, polymer coated aggregate, durability

## Abstract

Pumice aggregates with low density and high porosity are widely used in lightweight concrete. The high water retention ability of pumice aggregates adversely affects the properties of fresh concrete. Additionally, pumice aggregates’ inadequate mechanical strength and durability hinder concrete performance. In recent years, research on coated aggregates has gained traction to improve the physical properties, mechanical strength, and durability characteristics of concrete. In this study, coarse pumice aggregates were coated with polyester and partially substituted with uncoated aggregates at ratios of 0%, 25%, 50%, 75%, and 100% in lightweight concrete formulations. Specific weight and water absorption tests were performed on the aggregates, while slump and unit weight tests were performed on fresh concrete mixtures. SEM-EDX analyses, unit weight, water absorbing capacity, sorptivity, compressive strength, freeze-thaw resistance, and sulfate resistance tests were performed on concrete specimens. The results indicated that the polyester coating significantly increased the specific weight of the aggregates and decreased the water absorption rates by up to 85%. Despite the coated aggregates resulting in decreased compressive strength of concrete specimens, they demonstrated reduced water absorbing capacity and sorptivity characteristics relative to reference concrete. Moreover, concrete made with coated aggregates exhibited better results in freeze-thaw and sulphate resistance tests.

## 1. Introduction

Traditionally, concrete produced using natural coarse aggregates has unit weights mostly ranging between 2400–2500 kg/m^3^ [1,2]. One of the most significant disadvantages of conventional concrete is its high weight. This has made the reduction in structural weight a critical issue in the construction sector, drawing the attention of engineers and researchers. Lightweight concrete (LWC) is generally defined as concrete with an oven-dry density of less than 2000 kg/m^3^ [3]. According to the RILEM/CEB standards, lightweight concrete is classified as structural, structural/insulation, or insulation concrete based on properties such as compressive strength, thermal conductivity, and density [3]. Additionally, ACI 213R defines structural lightweight concrete as having a minimum 28-day compressive strength of 17 MPa and a density ranging from 1120 to 1920 kg/m^3^ [4]. Lightweight concrete is distinguished by several advantages, including the reduction of dead loads, decreased overall construction costs, reduced structural steel requirements, smaller foundation dimensions, low thermal conductivity, improved fire resistance, and effective insulation against heat and sound [5,6,7]. These features have promoted the widespread use of lightweight concrete in civil engineering applications.

The performance of concrete in the construction sector is largely dependent on the properties of its components. Aggregates, which constitute approximately 75% of concrete, are among the primary components that significantly influence its mechanical and physical performance. Since coarse aggregates are a major determinant of concrete density, replacing natural coarse aggregates with lightweight aggregates (LWA) is a common method for producing lightweight concrete. However, the high water absorption capacity of lightweight aggregates can adversely affect the properties of both fresh and hardened concrete. Due to their internal porosity and low density, lightweight aggregates can easily absorb mixing water or rise to the surface during mixing. This can lead to excessive slump variations in fresh concrete, difficulties in pumping, shrinkage issues, and cracking after drying [3,8,9,10]. Additionally, during the hydration process, the high water absorption capacity of lightweight aggregates can increase the amount of unhydrated cement in the interfacial transition zone (ITZ). This can negatively impact the mechanical strength and long-term durability properties of the concrete, resulting in a decline in structural performance [11]. To mitigate these disadvantages, appropriate mix design and water absorption control methods must be implemented.

Several methods are available for protecting lightweight aggregate concrete (LWAC) mixtures from workability loss and segregation. One of the most common methods involves pre-saturating lightweight aggregates (LWA) before their inclusion in the concrete mix. Alternatively, additional water corresponding to the absorption capacity of the LWA can be incorporated into the mix. In most cases, the aggregates are fully saturated before being added to the concrete. These practices are effective, simple, and economical methods for reducing the risks of workability loss and segregation and are widely applied in practice today. However, some adverse effects of aggregate pre-saturation have also been reported. In concrete containing LWA with high water absorption capacity, pre-saturation or high moisture content has been associated with reduced mechanical strength [12,13], increased water permeability [12,14], lower freeze-thaw resistance [15,16], deeper carbonation [17,18], and greater penetration of chloride ions [17,18]. Moreover, studies on the microstructure of LWAC have shown that LWAC prepared with pre-saturated or highly moistened aggregates exhibits a weaker bond structure in the interfacial transition zone (ITZ) due to increased portlandite crystals, higher ettringite content, and additional microcracks [14,19]. For these reasons, researchers have focused on alternative approaches, such as coating lightweight aggregates, to mitigate these disadvantages.

In recent years, new methods have been explored to enhance the physical and mechanical properties as well as the durability characteristics of lightweight aggregate concretes. Among these methods, impregnating high-porosity lightweight aggregates with cement paste or coating them with cement paste are notable examples [10,14,20]. However, the cement impregnation technique augments the aggregate mass, consequently undermining one of the principal benefits of lightweight aggregates, which is their lower unit weight. Additionally, the lengthy application procedure of this method is a significant drawback. Utilization of polymers for the modification of aggregates has emerged as a promising alternative due to their ability to limit the increase in unit weight and offer a more practical application process. Although studies involving polymer-coated aggregates are limited in the literature, they have been shown to yield positive results [8,9,21,22]. A comprehensive literature review indicates that there are only a few studies on the polymer coating of natural lightweight aggregates, such as pumice. However, it appears that no detailed study focusing on the durability properties of polymer-coated aggregates has been conducted.

Pumice aggregate (PA) is a natural lightweight aggregate formed by the rapid cooling of erupting liquid volcanic material. Due to its volcanic origin, PA is rich in silica content. Pumice is an exceptionally lightweight rock material owing to its cellular structure. This cellular structure consists of independent air pockets formed as gases escape from liquid magma during cooling. Because of its low density and other advantageous properties, pumice aggregate is widely used as a coarse aggregate in the production of lightweight concrete worldwide [1,23]. These characteristics make PA an ideal alternative both as a construction material and for enhancing the density and insulation performance of concrete.

In recent years, the number of studies focusing on the coating of lightweight aggregates has increased significantly. In one study, pumice aggregates were coated with three different polymers, and lightweight concrete mixtures were produced with three cement dosages (300/400/500 kg/m^3^). The study reported that the dry unit weights of the produced concretes ranged between 928 and 1341 kg/m^3^, while their 28-day compressive strengths were between 5.5 and 17.3 MPa. The compressive strength analysis revealed that specimens prepared with aggregates coated using a polyurethane-based polymer (designated as KBP) exhibited higher strength values compared to reference specimens across all cement dosages [24]. Salli Bideci et al. (2014) [25] further reported that they coated pumice aggregates with three different polymers. They observed that the specific gravity of coated and uncoated lightweight aggregates ranged from 0.98 to 1.64 g/cm^3^. Additionally, they noted that the specific gravity values of aggregates with pore sizes of 4–8 mm and 8–16 mm increased with the polymer coating compared to the control group. According to these findings, pumice aggregates with high water absorption rates (30–40%) can be transformed into lightweight pumice aggregates with low water absorption rates (2–10%) through the polymer coating method.

In a study where coarse pumice aggregates were coated with a mixture of cement and colemanite (0%, 7.5%, 12.5%, 17.5%) for the production of lightweight concrete, the resulting concrete specimens were exposed to varying temperatures of 20 °C, 200 °C, 400 °C, and 600 °C. The results showed that specimens coated with a mixture containing 12.5% colemanite achieved optimum performance [26]. In one study, the surfaces of three different lightweight aggregates were coated with the cement paste impregnation method, and concrete specimens were produced using the aggregates obtained. It was observed that the cement paste-impregnated aggregates had a 1.8–3.7 times lower water absorption rate compared to normal aggregates. They determined that the unit weight increased by up to 19%, mechanical strength increased by up to 107%, and water absorption rates decreased by up to 52% in concretes produced with these aggregates [10].

Vahabi et al. (2022) [9] utilized PVA and SBR latex polymers to coat scoria and leca aggregates for lightweight concrete production. As a result of the study, they reported an increase of up to 10% in the flowability of concrete mixtures produced with coated aggregates. They also reported that the water absorption rates of lightweight aggregates decreased by 80–90% after coating, the water absorption rates of concrete samples decreased by up to 29%, and there was a 21–24% increase in compressive strength. Akyüncü and Şanlıtürk (2021) [22] developed new aggregates by coating expanded perlite with polymers and used these aggregates in mortar mixtures at 20%, 40%, 60% and 80% as a substitute for natural aggregates. The use of perlite aggregates caused a decrease in unit weight, compressive strength, and ultrasonic impact velocity, as well as an increase in water absorption. Mortars produced with coated aggregates showed less water absorption and sorptivity than those produced with uncoated aggregates.

Özgüler et al. (2023) [20] coated coarse pumice aggregates obtained from two different sources with cement paste. Using a scanning electron microscope (SEM), they examined the coating thickness, which was found to be between 550 μm and 1060 μm. They observed a reduction in pulverization of approximately 16% and 19%, respectively, based on the impact value test. Additionally, the water absorption rates decreased by 50.49% and 78.32%, respectively. Bideci et al. (2023) [8] coated pumice aggregates with polyester resin and produced lightweight concrete samples using these coated aggregates. In this study, coated aggregates were used to replace uncoated aggregates at ratios of 0%, 50%, and 100% with three different cement dosages. The results showed that the polyester coating process increased the specific gravity of the aggregates and reduced the water absorption rates by up to 95%. In the concrete samples, the use of coated aggregates led to an increase in unit weights and a reduction in water absorption rates between 23.9% and 54.2%. However, it was noted that the use of coated aggregates resulted in a decrease in the compressive strength of the concrete samples.

A review of the literature reveals numerous studies focusing on the modification of lightweight aggregates using coating methods. However, it has been observed that detailed investigations specifically addressing the utilization of polymer-coated pumice aggregates remain limited. In this study it is aimed to develop a new lightweight aggregate with reduced water absorption capacity by coating natural pumice aggregates, which adversely affect both fresh and hardened concrete properties due to their high water absorption properties, and to produce lightweight concrete with high durability using coated aggregates. In this study, pumice aggregates with particle sizes ranging from 4 to 16 mm were coated with polyester to produce modified lightweight aggregates. Subsequently, five series of structural lightweight concrete specimens were prepared with a cement dosage of 450 kg/m^3^. The concrete mixture compositions contain crushed aggregates (0–4 mm), along with coated and uncoated pumice aggregates ranging from (4 to 16 mm). The properties of fresh concrete were evaluated using unit weight and slump tests. The hardened concrete specimens were assessed for unit weight, water absorption, sorptivity, compressive strength at 28 and 90 days, freeze-thaw resistance, and sulfate resistance. Additionally, SEM-EDX analyses were conducted to examine the microstructural characteristics, providing comprehensive insights into the performance of the developed concrete.

## 2. Materials and Methods

### 2.1. Materials

In this study, pumice aggregates with particle sizes of 4–8 mm and 8–16 mm were used as coarse aggregates, both in their polyester-coated and uncoated forms. Crushed natural aggregates with particle sizes of 0–4 mm were used as the fine aggregates. CEM I 42.5/R cement conforming to TS EN 197-1 [27] standards was utilized as the binder. Marble powder with a particle size of 100 μm was used to separate the aggregates after the polyester coating process. Table 1 presents the results of the chemical analyses of mineral-based materials.

The polyester utilized in this study is an unsaturated cast-based polyester resin. Polyester is commonly utilized in filled casting applications, such as artificial marble, owing to its favorable filler acceptance and minimal shrinkage characteristics. It is particularly suited for scenarios where rapid curing and elevated thermal resistance are not essential. The properties related to the physical and mechanical aspects of the polyester utilized are detailed in Table 2 [28].

### 2.2. Polyester Coating Procedure of Pumice Aggregates

**Figure 1:**
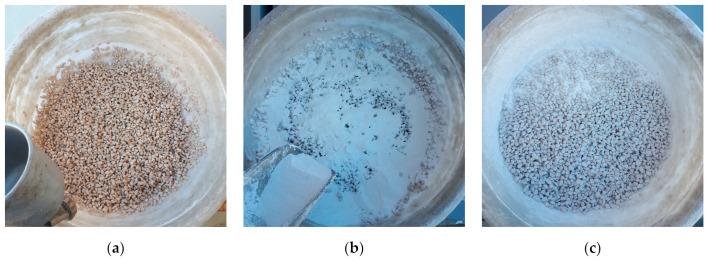
Polyester coating process: (**a**) Polyester spraying process on aggregates; (**b**) Marble dust sprinkling process to separate aggregates; (**c**) Complete coating and separation of aggregates.

**Figure 2:**
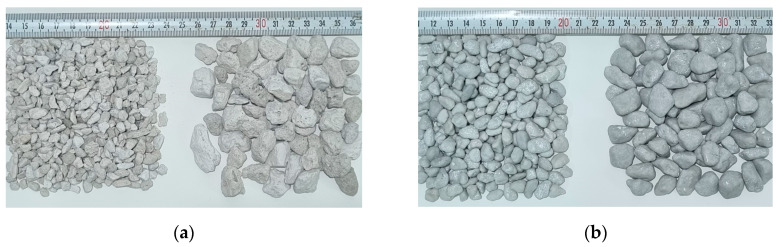
Pumice aggregates: (**a**) Uncoated pumice aggregates; (**b**) Coated pumice aggregates.

Pumice aggregates with particle sizes in the ranges of 4–8 mm and 8–16 mm were dried in an oven at 100 °C for 24 h and subsequently cooled at room temperature for 2 h prior to the polyester coating process. The coating process was performed using a spray method with a top-feed paint spray gun having a nozzle diameter of 0.8–2.8 mm and a compressor generating air pressure of 6–8 bar. The dense consistency of the polyester was diluted with a 6% cellulosic thinner to facilitate spraying with the paint gun. During the coating process, approximately 2 kg of aggregates were coated with polyester in each batch using plastic containers with a diameter of 40 cm and a depth of 25 cm. The polyester-coated aggregates were left to cure at a temperature of 23 ± 2 °C for 96 h. This process was repeated three times, resulting in a total of three layers of polyester coating on the pumice aggregates. The polyester coating process is illustrated in Figure 1, and the coated and uncoated pumice aggregates are shown in Figure 2.

### 2.3. Mixtures Proportions of Lightweight Concrete

**Figure 3:**
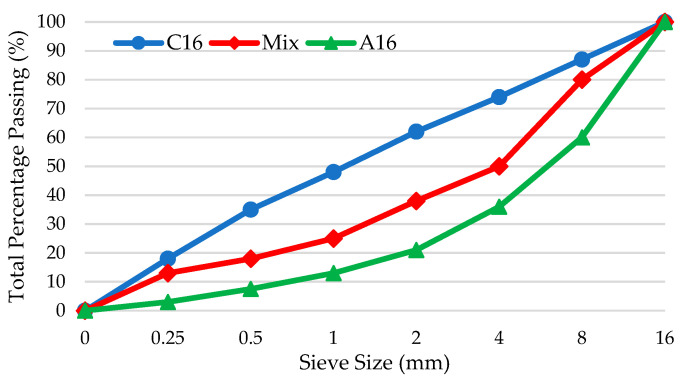
Sieve analysis of aggregates.

In the lightweight concrete mixtures, the cement content was fixed at 450 kg/m^3^, and the water-to-binder ratio was maintained at 0.5. The total aggregate volume consisted of 50% fine aggregates with particle sizes of 0–4 mm, 30% coarse aggregates with particle sizes of 4–8 mm, and 20% coarse aggregates with particle sizes of 8–16 mm. The results of the aggregate sieve analysis are presented in Figure 3. Coated pumice aggregates were substituted for uncoated aggregates at volumetric replacement ratios of 25%, 50%, 75%, and 100%, and a control series composed entirely of uncoated aggregates was also prepared. The proportions of the produced lightweight concrete mixtures are presented in Table 3.

### 2.4. Methods

The specific gravity tests of the aggregates and the determination of water absorption rates for coated and uncoated aggregates were conducted in accordance with the provisions specified in the TS EN 1097-6 [31] standard. To evaluate the workability of fresh concrete, a slump test was performed in compliance with the TS EN 12350-2 [32] standard, and a unit weight test was carried out following the TS EN 12350-6 [33] standard.

**Figure 4:**
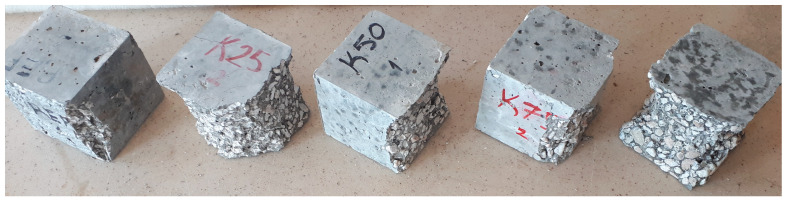
Concrete samples after compressive strength test.

In this study, hardened concrete tests were conducted on cube specimens with dimensions of 100 × 100 × 100 mm. The dry unit weight and water absorption rates were determined in accordance with the TS EN 12390-7 [34] standard, while the compressive strengths were measured following the TS EN 12390-3 [35] standard. The specimens after the compressive strength test are presented in Figure 4.

The sorptivity coefficients of the concrete specimens were determined experimentally in accordance with the ASTM C1585 [36] standard. The lateral surfaces of the concrete specimens were coated with a polymer, leaving the parts immersed in water. Prior to the test, the specimens were dried in an oven at a temperature of 100 ± 5°C for 24 h, and their initial weights were recorded. Subsequently, their weights were measured at specified intervals to calculate the sorptivity coefficients.

In the freeze-thaw test, 30 cube specimens with dimensions of 100 × 100 × 100 mm were used. After being cured in water for 28 days, the concrete specimens were placed in a freeze-thaw apparatus for 50 and 100 freeze-thaw cycles. Each freeze-thaw cycle was conducted in the apparatus, completing one cycle within a 6-h period, with temperatures ranging from +20 °C to −20 °C.

**Figure 5:**
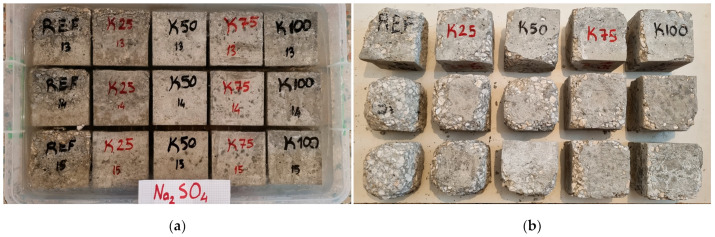
Resistance test to sodium sulphate: (**a**) Specimens immersed in the Na_2_SO_4_ solution; (**b**) Specimens after being immersed in the Na_2_SO_4_ solution.

For the sulfate attack test, 30 concrete cube specimens with dimensions of 100 × 100 × 100 mm were used after completing a 28-day water curing period. The specimens were dried in an oven at 100 ± 5 °C, and their dry weights were recorded. Sulfate solutions were prepared as mixtures containing 25% sodium sulfate (Na_2_SO_4_) and magnesium sulfate (MgSO_4_). The specimens were immersed in the solutions for 24 h, then dried in an oven for another 24 h, and their weights were measured again. This procedure was repeated 10 times. At the end of the test, the weight loss was measured for the specimens exposed to the Na_2_SO_4_ solution, while the compressive strength was measured for those exposed to the MgSO_4_ solution. The visuals of the specimens exposed to the Na_2_SO_4_ solution during the test process are shown in Figure 5.

Scanning Electron Microscopy (SEM) and secondary electron imaging of the concrete samples were conducted using the EDASDD Apollo 40 apparatus.

## 3. Results

### 3.1. Aggregate Test Results

This section may be divided by subheadings. It should provide a concise and precise description of the experimental results, their interpretation, and the experimental conclusions that can be drawn. The specific gravities of the aggregates, determined based on the volumetric displacement of water by the aggregates, and their water absorption rates, calculated relative to their oven-dry weights, are detailed in Table 4.

The specific gravities of the aggregates were observed to range between 0.92 and 2.65 g/cm^3^. It was noted that the specific gravities of pumice aggregates increased after the polyester coating process. This phenomenon was attributed to the higher specific gravity of the polyester used in the coating process compared to that of the pumice aggregate. Previous literature reports indicate that pumice aggregates typically exhibit water absorption rates ranging from 30% to 40%, which decrease to 2–10% with various polymer coatings [8,25]. Uncoated pumice aggregates with a particle size of 4–8 mm used in lightweight concrete production exhibited a water absorption rate of 29.3%, whereas polyester-coated pumice aggregates with the same particle size demonstrated a significantly reduced water absorption rate of 4.4%. Similarly, for pumice aggregates with particle sizes of 8–16 mm, the water absorption rate was 32.1% for the uncoated aggregates and 4.8% for the polyester-coated aggregates. Notably, the water absorption rates of the pumice aggregates decreased by a factor of 5–6 after the polyester coating process. This observation suggests that the application of polyester coating holds promise for enhancing the impermeability properties of pumice by significantly reducing its water absorption capacity.

### 3.2. Concrete Test Results

The unit weights and slump levels of the fresh concrete are given in Table 5. The unit weights of the fresh concrete samples were determined to range between 1766 kg/m^3^ and 1907 kg/m^3^. The lowest unit weight of 1766 kg/m^3^ was observed in the REF series of concrete samples, in which all pumice aggregates were uncoated. Compared to the REF samples, it was found that the unit weights increased as the amount of polyester-coated pumice aggregates in other series increased. The slump values of the fresh concrete samples were found to range from 11 mm to 114 mm. The lowest slump value of 11 mm was measured in the REF series, which consisted of lightweight concrete without polyester-coated pumice. The use of a fixed water-to-binder ratio across all concrete mixtures, along with the high water absorption capacity of uncoated pumice aggregates and the low water absorption capacity of polyester-coated pumice aggregates, resulted in differences in workability. It has been observed that the workability of concrete mixtures increases with the proportion of polyester-coated aggregates. As expected, this improvement is attributed to the high water absorption capacity of the uncoated pumice aggregates, which absorb a portion of the mixing water.

Dry unit weights, water absorption rates, 28 and 90 day compressive strengths, and sorptivity values of the hardened concrete specimens are given in Table 6. When the dry unit weight results of the concrete samples were examined, it was observed that the highest unit weight, measured at 1825.5 kg/m^3^, occurred in the PC-100 series specimens. It has been determined that, within these concrete series, the dry unit weight values are directly related to the proportion of coated pumice aggregate in the concrete, and that the unit weight of the concrete increases as the coated pumice ratio rises. In terms of the dry unit weight, compared with the REF series specimens, increases of 2.4%, 3.7%, 4.6%, and 6.6% were observed in the PC-25, PC-50, PC-75, and PC-100 series, respectively.

When examining the water absorption rates of the concrete series, they were found to vary between 5.0% and 6.3%. The highest water absorption rate (6.3%) was observed in the REF-coded concrete series, while the lowest rate (5.0%) was recorded for the PC-50-coded series. In all concrete series incorporating polyester-coated pumice aggregates, the water absorption rate decreased compared to the control series without polyester-coated pumice. In particular, the PC-50 series exhibited a 20% reduction in water absorption relative to the REF series, indicating that the polyester coating on the aggregate contributed to this decrease. However, because the same amount of water was used in all the series and the uncoated aggregates absorbed a portion of the mixing water, thereby indirectly affecting the water/binder ratio, no strictly linear reduction in water absorption was observed for the series containing polyester-coated aggregates. In other words, the indirectly altered water-to-binder ratios of the concrete mixtures also influenced their permeability properties [37]. Although coated aggregates typically exhibit lower water absorption rates, the higher water absorption observed in the PC-75 and PC-100 series concrete specimens compared to the PC-50 series provides supporting evidence for this phenomenon. Despite this, the findings of this study are in agreement with the water absorption ranges reported for lightweight concrete in the literature, which typically lie between 6% and 12% [38,39]. Moreover, they satisfy the requirement suggested by Neville (1995) and Aitcin (1998) [40,41], who indicated that the maximum water absorption for high-durability lightweight concretes can be as high as 10%.

**Figure 6:**
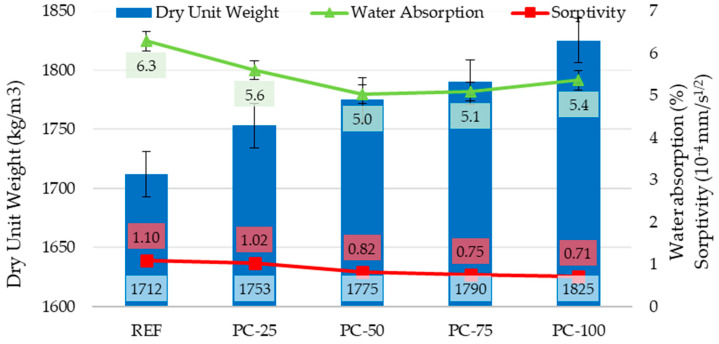
Dry unit weights, water absorption rates, and sorptivity coefficients of hardened concretes.

Sorptivity is directly associated with the capillary pores present in the concrete mortar. In concretes produced with lightweight aggregates, the sorptivity coefficient is lower compared to those containing normal-weight aggregates, owing to the elastic compatibility and strong mechanical bond formed at the aggregate–matrix interface [17,42]. Geçten and Gül (2013) [43] reported that the coefficients of sorptivity for 28-day-old concrete specimens produced with normal-weight and lightweight aggregates were 2.18 × 10^−4^ cm^2^/min and 1.30 × 10^−4^ cm^2^/min, respectively. Examination of the present data revealed that the sorptivity coefficients of the concrete specimens ranged from 1.10 × 10^−4^ cm/s^0.5^ to 0.71 × 10^−4^ cm/s^0.5^. The highest value was obtained for the REF series specimens. Relative to the REF series, the sorptivity coefficients decreased by 7% in the PC-25 series, 25% in the PC-50 series, 32% in the PC-75 series, and 36% in the PC-100 series. The dry unit weights, water absorption rates, and sorptivity coefficients of the concrete specimens are presented in Figure 6.

**Figure 7:**
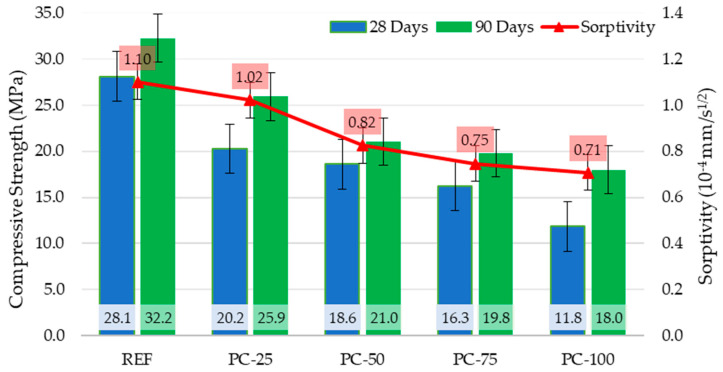
28–90-day compressive strengths and sorptivity coefficients of concrete specimens.

Figure 7 shows the 28–90-day compressive strengths and sorptivity coefficients of the concrete specimens. In the context of 28-day compressive strength evaluations of the concrete specimens, the highest strength value of 28.1 MPa was recorded in the REF series specimens, whereas the lowest value of 11.8 MPa was observed in the PC-100 series specimens. A linear reduction in the compressive strength was noted as the proportion of the coated pumice aggregate in the concrete mixture increased. Relative to the REF control series, the compressive strengths of PC-25, PC-50, PC-75, and PC-100 series decreased by 28%, 34%, 43%, and 58%, respectively. Similarly, based on the 90-day compressive strength results, the highest strength of 32.2 MPa was obtained from the REF series specimens, while the lowest strength of 18 MPa was measured in the PC-100 series. Compared with the REF series, the 90-day compressive strengths of the PC-25, PC-50, PC-75, and PC-100 series were reduced by 20%, 35%, 39%, and 44%, respectively.

When comparing the 28-day and 90-day compressive strengths, all concrete series exhibited an increase in compressive strength at 90 days. Among these, the PC-100 series specimens demonstrated the highest increase, with a 52% improvement in the 90-day compressive strength relative to their 28-day strength. This was followed by the PC-25 series with a 28% increase, PC-75 series with a 22% increase, REF series with a 15% increase, and PC-50 series with a 13% increase. Considering these results, it can be inferred that the use of polyester-coated aggregates may delay the process of achieving the final strength. Moreover, the lower rate of strength gain observed in the PC-50 series compared to the REF series has been attributed to the heterogeneous nature of the concrete.

According to the TS EN 206+A2 [44] standard, the compressive strength of lightweight concrete ranges between 9 and 88 MPa for 28-day cube specimens and 8 and 80 MPa for 28-day cylindrical specimens. An analysis of the experimental data indicates that the 28-day compressive strength values of the concrete specimens fall within the range of 28.1 MPa to 11.8 MPa. Based on this observation, it is determined that the produced concrete series comply with the standard, with their compressive strength classes identified as LC 25/28 for the REF series, LC 16/18 for the PC-25 and PC-50 series, LC 12/13 for the PC-75 series, and LC 8/9 for the PC-100 series. Among these series, the 28-day compressive strengths of the REF, PC-25, and PC-50 specimens meet the criterion of 17 MPa for structural lightweight concrete, as specified in the literature. However, the 28-day compressive strengths of the PC-75 and PC-100 specimens fall below this threshold. Nonetheless, an evaluation of the 90-day compressive strengths reveals that all concrete series achieve compressive strengths exceeding 17 MPa, thereby fulfilling the requirements for structural lightweight concrete at this age.

Adverse environmental conditions, such as freeze-thaw cycles, are well-known to detrimentally affect the durability of concrete, leading to structural damage and diminished overall performance. In the present study, the concrete specimens produced were subjected to two different freeze-thaw cycles, 50 and 100 cycles, to assess their resistance under these conditions. After completion of the freeze-thaw processes, no visible fractures or mass losses were observed in the specimens; subsequently, their compressive strengths were measured.

After 50 freeze-thaw cycles, the highest compressive strength was observed in the REF series specimens (29.1 MPa), while the lowest was recorded in the PC-100 series specimens (12.5 MPa). A comparison of the compressive strengths before and after 50 cycles revealed an increase in some specimens and a decrease in others. Specifically, the compressive strength increased by 3.4% in REF specimens, 4.6% in PC-25 specimens, and 5.8% in PC-100 specimens. In contrast, a decrease of 3.9% was observed in the PC-50 specimens, while the compressive strength of the PC-75 specimens remained unchanged. These limited variations in compressive strength are hypothesized to be more related to the heterogeneous structure of the concrete rather than the effects of freeze-thaw cycles themselves.

**Figure 8:**
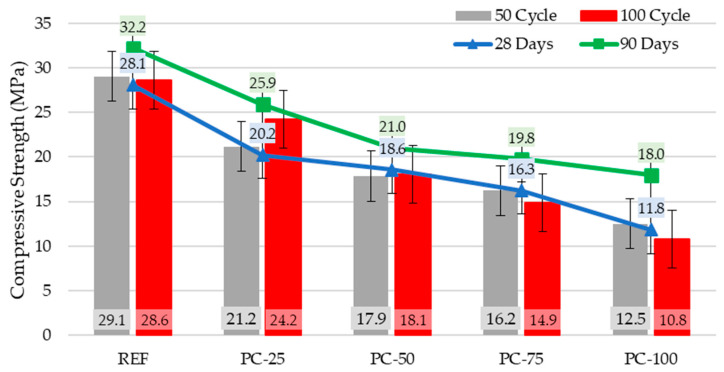
Compressive strength of specimens before and after freezing and thawing cycles.

A comparison of the 28-day compressive strengths of the concrete specimens with their compressive strengths after 100 freeze-thaw cycles revealed varying trends among the series. The REF series exhibited a 1.9% increase, while the PC-25 series showed a significant increase of 19.7%. In contrast, the compressive strengths of the PC-50, PC-75, and PC-100 series decreased by 2.8%, 8.6%, and 8.9%, respectively. Based on the 100 freeze-thaw cycle results, all polyester-coated aggregate concrete series, except for the PC-25 series, experienced a reduction in compressive strength. Previous studies have suggested that incorporating lightweight aggregates into concrete at specific proportions can enhance the freeze-thaw resistance of concrete. Moreover, the observed increase in compressive strength after freeze-thaw cycles may be attributed to the escape of excess water into the pores of lightweight aggregates during freezing events [45,46]. Among the concrete mixtures, the reference series and the PC-25 series, featuring the lowest proportion of coated aggregates, exhibited the highest performance in terms of freeze–thaw resistance. This outcome has been attributed to the migration of water into the aggregate pores during the freezing process. Furthermore, the increase in compressive strength observed in the PC-25 series after freeze–thaw cycles has been associated with the heterogeneous nature of the concrete. The polyester coating on the aggregates effectively reduced their water absorption, thereby minimizing the potential for water infiltration into the aggregate voids. Considering this, the reductions in compressive strength observed for the PC-50, PC-75, and PC-100 series after 100 freeze-thaw cycles were 2.8%, 8.6%, and 8.9%, respectively, and this can be explained by the interplay between the reduced water absorption of coated aggregates and the limitations imposed by their heterogeneous structure. The compressive strengths of the concrete specimens before and after freezing and thawing are shown in Figure 8.

Mehta and Monteiro (2014) [47] stated that due to a sulfate attack on the cement matrix in concrete, the C-S-H gel breaks down, which has a negative effect on concrete. In this study, two different sulfate solutions (MgSO_4_ and Na_2_SO_4_) were prepared to evaluate the resistance of the produced lightweight concrete specimens to sulfate attack. The specimens immersed in the Na_2_SO_4_ solution exhibited notable fragmentation and deterioration. For comparative purposes, the mass losses of the specimens were measured. Since there was a limited mass loss in the specimens immersed in MgSO_4_ solution, the compressive strengths were tested after the specimens were kept in the sulfate solution. In the majority of studies in the literature, magnesium sulfate environments have been reported to be more detrimental to concrete than sodium sulfate environments [48,49]. However, in some investigations focusing on the effects of high concentrations of magnesium and sodium sulfates on concrete durability, sodium sulfate environments have been shown to lead to greater mass loss compared with magnesium sulfate environments [50,51]. The mass losses of the specimens immersed in the Na_2_SO_4_ and MgSO_4_ solutions are given in Table 7.

**Figure 9:**
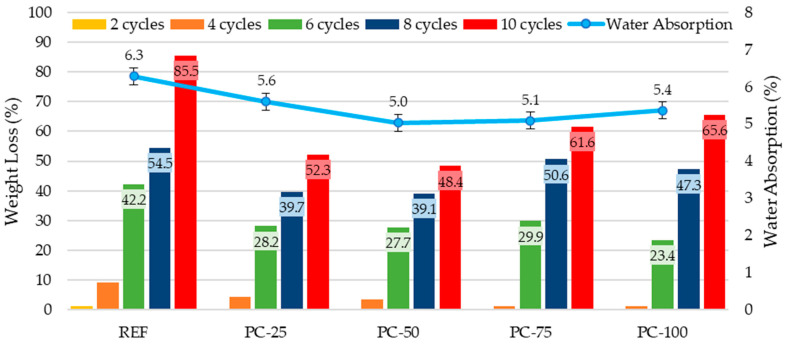
Water absorption rates of the samples and mass losses after immersion in Na_2_SO_4_ solution.

An analysis of the mass losses of specimens immersed in the Na_2_SO_4_ solution revealed that the highest mass loss occurred in the REF series specimens, with a loss of 85.5%, while the lowest was observed in the PC-50 series specimens, with a loss of 48.4%. The mass losses of the other specimens were recorded as 52.3% for the PC-25 series, 61.6% for the PC-75 series, and 65.6% for the PC-100 series. A direct correlation was identified between the water absorption rates of the concrete specimens and their mass losses after exposure to the Na_2_SO_4_ solution. All concrete specimens containing polyester-coated aggregates exhibited significantly lower mass losses compared to the REF series control specimens. This indicates the beneficial effect of the polyester coating in reducing sulfate-induced deterioration. The water absorption rates of the samples and mass losses after immersion in the Na_2_SO_4_ solution are presented in Figure 9.

**Figure 10:**
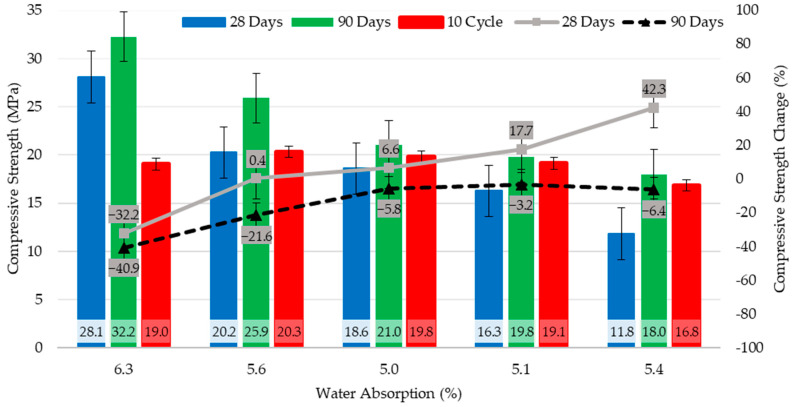
Water absorption rates of the samples, compressive strength of 28 and 90 days, compressive strength after immersion in MgSO_4_ solution and change of compressive strength after immersion in MgSO_4_ solution.

When the percentage mass losses of the specimens immersed in MgSO_4_ solution were analyzed, it was observed that the highest mass loss among all specimens was in the PC-25 series specimens with 2.4%, and the lowest mass loss was in the PC-100 series specimens with 1.9%. Some differences were observed in the compressive strength values of the concrete specimens after immersion in the MgSO_4_ solution. Compared to the 28-day compressive strength specimens, the PC-25 series increased by 0.4%, the PC-50 series increased by 6.6%, the PC-75 series increased by 17.7%, and the PC-100 series increased by 42.3%, while REF-coded control series decreased by 32.2%. The water absorption rates of the samples, compressive strengths at 28 and 90 days, compressive strengths after immersion in MgSO_4_ solution, and changes in compressive strength after immersion in MgSO_4_ solution are shown in Figure 10.

The SEM-EDS analyses reveal that the samples consist of lightweight aggregate particles with mineral content embedded in a glassy structure [8]. It has been reported that the pore periphery of pumice aggregates contains micron-sized tubular channels and exhibits high porosity [52,53]. The porosity and permeability properties of concrete are largely dependent on the interfacial transition zone (ITZ) characteristics, and microcracks within the matrix phase are known to influence these properties [54]. Moreover, the pore structure of concrete plays a significant role in its strength, with physical properties such as porosity and compactness directly correlated with the mechanical properties of the material. Marble dust used in the coating material has the potential to alter the pore structure of concrete [55].

**Figure 11:**
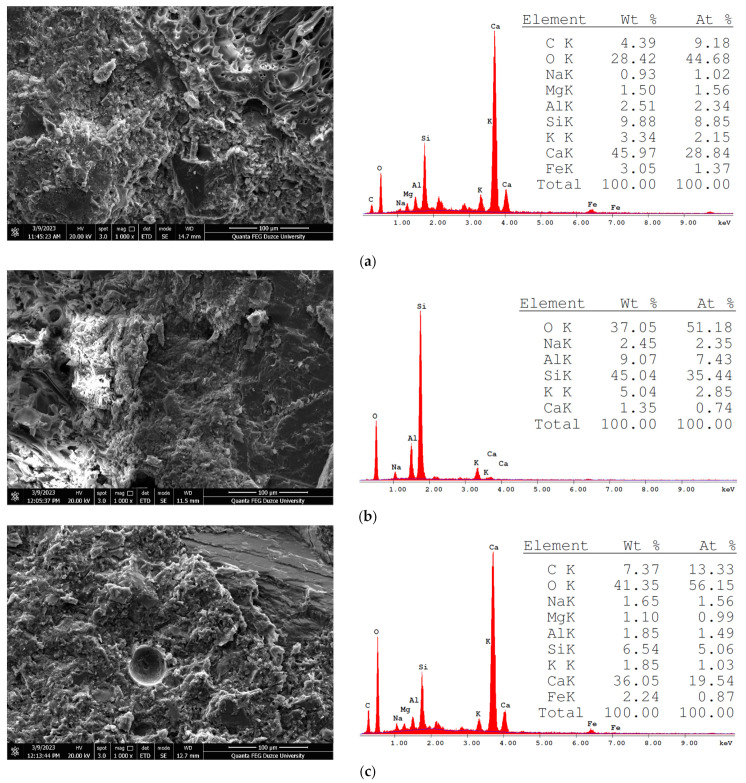
SEM-EDS images of lightweight concrete: (**a**) REF series; (**b**) PC-50 series; (**c**) PC-100 series.

In the analysis of the REF series sample, the dominant peak observed in the EDS results indicates that the sampled region is primarily located in the interfacial transition zone (ITZ), which is rich in cement paste or hydration products, such as portlandite (CH) and C-S-H phases. A high Ca-to-Si ratio is characteristic of areas closer to cement hydrates rather than pumice aggregates. Under SEM, a relatively porous microstructure is observed, with open channels or voids associated with the rough surface texture and intrinsic porosity of natural pumice. For the PC-50 series, the EDS results show high Si and low Ca peaks, suggesting that the analyzed area is closer to the pumice aggregate or a transition zone significantly influenced by either coated or uncoated pumice. This indicates that the SEM images in this series have mixed characteristics of both the aggregate and cement paste. In the PC-100 sample, the EDS results reveal peaks partially resembling those of the REF sample, suggesting that the analyzed region is likely the ITZ rather than the polyester-coated aggregate itself. Under SEM, the ITZ regions appear to include smoother surfaces from the polyester-coated aggregates alongside rougher surfaces from natural pumice. This indicates reduced visible pore throats in the coated regions, contributing to the densification of the microstructure in the ITZ. From the SEM-EDS analyses, the characteristic elemental peaks corresponding to C-S-H, portlandite, ettringite, unhydrated cement particles, and marble dust were identified. SEM-EDS images of the lightweight concrete specimens obtained with polyester-coated and uncoated pumice aggregates are presented in Figure 11.

## 4. Discussion

In this study, lightweight concrete was produced using both uncoated and polyester-coated pumice aggregates with a cement dosage of 450 kg/m^3^, and their fresh and hardened properties were comprehensively evaluated.

The polyester coating treatment resulted in an approximately 3–8% increase in the aggregate unit weight and yielded a substantial reduction on the order of 85% in water absorption.

Specifically, the water absorption values of the coated aggregates ranged from 4.4% in the 4–8 mm size fraction to 4.8% in the 8–16 mm size fraction.

Despite maintaining a constant water-to-binder ratio (w/b) in all concrete mixtures, the slump values of fresh concrete showed marked variability (11–114 mm) when uncoated aggregates were used, mainly due to their high water absorption capacity.

The dry unit weights of the concrete incorporating both coated and uncoated pumice aggregates were between 1712 and 1826 kg/m^3^, thus remaining below the 2000 kg/m^3^ threshold commonly adopted for lightweight concrete applications.

The water absorption values of the hardened concrete varied in the 5–6% range, with all samples produced using coated aggregates demonstrating lower absorption compared to the control sample.

The sorptivity coefficients of the produced concrete were found to range between 0.71 × 10^−4^ and 1.10 × 10^−4^. The highest sorptivity coefficient was observed for the REF series concrete specimens. Moreover, as the proportion of polyester-coated pumice aggregates in the concrete mixtures increased, a corresponding decrease in the capillary water absorption coefficient was observed.

The highest compressive strengths were obtained in the reference samples (REF) containing uncoated aggregates, while the use of polyester-coated aggregates resulted in a reduction in compressive strength. However, in comparison to the 28-day compressive strength, the 90-day compressive strength demonstrated the highest increase in the samples produced with polyester-coated aggregates. This phenomenon is attributed to the reduced water absorption capacity of the coated aggregates, which indirectly affects the effective water-to-binder (w/b) ratio in the concrete mix, thereby influencing the strength development over time.

Based on the compressive strength data obtained after freeze-thaw cycles, a maximum change of 5% in strength values was observed after 50 cycles. However, after 100 cycles, specimens containing a higher proportion of coated aggregates exhibited lower freeze-thaw resistance.

Specimens produced with polyester-coated aggregates demonstrated enhanced resistance to sulfate attack, which was attributed to the lower water absorption properties of the coated aggregates.

In the SEM-EDS analyses, lightweight aggregates exhibiting a glassy structure comprised of porous minerals and hydrated phases, including C-S-H, portlandite, ettringite, and non-hydrated cement particles, were detected.

In conclusion, the use of polymer-coated pumice aggregates as replacements for concrete has led to significant improvements in certain durability properties. To enable a more comprehensive analysis of polymer-coated pumice aggregates, future studies could focus on evaluating the impact resistance of aggregates, as well as conducting high-temperature resistance and more extensive freeze-thaw cycles on concrete specimens.

## Figures and Tables

**Table 1:** Chemical analysis of the mineral-based materials (%).

Chemical Composition	SiO_2_	Al_2_O_3_	Fe_2_O_3_	CaO	MgO	K_2_O	Na_2_O	SO_3_	Loss Ignition
Pumice	66.80	14.75	2.85	2.95	0.80	2.75	3.95	-	4.15
CEM I 42.5/R	19.50	5.08	2.54	63.75	1.18	0.74	0.26	3.13	3.82
Marble Powder	-	-	0.02	31.16	23.31	0.01	0.51	-	44.89

**Table 2:** Technical properties of polyester.

Material Structure	Elongation at Break, Tensile	Elongation at Break, Flexural	Flexural Strength	Water Absorption	Color	Density (g/cm^3^)
Test Method	ISO 0178 [29]			ISO 0062 [30]	-	-
Polyester Modified Bitumen	% 2.50	% 5.94	138 MPa	%0.16	≤100 Hazen	1.125

**Table 3:** Lightweight concrete mixture proportions (kg/m^3^).

Series	Aggregates Particle Size (mm)					Cement	Water
	Crushed Sand	Pumice					
	0–4	4–8		8–16			
		Coated	Uncoated	Coated	Uncoated		
REF	834.5	0.0	175.7	0.0	114.6	450	225
PC-25	834.5	63.8	131.8	37.2	86.0	450	225
PC-50	834.5	127.5	87.9	74.3	57.3	450	225
PC-75	834.5	191.3	43.9	111.5	28.7	450	225
PC-100	834.5	255.1	0.0	148.6	0.0	450	225

**Table 4:** Specific gravities and water absorption rates of aggregates.

Aggregates	Sand	Pumice			
		Uncoated		Coated	
	0–4 mm	4–8 mm	8–16 mm	4–8 mm	8–16 mm
Specific gravity (g/cm^3^)	2.65	0.93	0.91	1.35	1.18
Water absorption (%)	1.6	29.3	32.1	4.4	4.8

**Table 5:** Slump and unit weight of fresh concretes.

Series	Slump (mm)	Unit Weight (kg/m^3^)
REF	11	1766
PC-25	19	1829
PC-50	32	1847
PC-75	70	1871
PC-100	114	1907

**Table 6:** Dry unit weights, water absorption rates, 28 and 90 day compressive and sorptivity values strengths of hardened concrete.

Series	Dry Unit Weight (kg/m^3^)	Water Absorption (%)	Compressive Strength (MPa)		Sorptivity (mm/s^1/2^)
			28 Days	90 Days	
REF	1712	6.3	28.1	32.2	1.10 × 10^−4^
PC-25	1753	5.6	20.2	25.9	1.02 × 10^−4^
PC-50	1775	5.0	18.6	21.0	0.82 × 10^−4^
PC-75	1790	5.1	16.3	19.8	0.75 × 10^−4^
PC-100	1825	5.4	11.8	18.0	0.71 × 10^−4^

**Table 7:** Mass losses of samples immersed in Na_2_SO_4_ and MgSO_4_ solutions.

Chemicals	Series	Weight Loss (%)				
		2 Cycles	4 Cycles	6 Cycles	8 Cycles	10 Cycles
Na_2_SO_4_	REF	1.3	9.3	42.2	54.5	85.5
	PC-25	0.3	4.5	28.2	39.7	52.3
	PC-50	0.4	3.5	27.7	39.1	48.4
	PC-75	0.4	1.2	29.9	50.6	61.6
	PC-100	0.2	1.1	23.4	47.3	65.6
MgSO_4_	REF	0.8	1.5	1.9	2.1	2.2
	PC-25	0.8	1.5	1.9	2.2	2.4
	PC-50	0.8	1.5	1.8	2.1	2.2
	PC-75	0.7	1.4	1.7	2.0	2.2
	PC-100	0.7	1.3	1.5	1.8	1.9

## Data Availability

Data are contained within the article.
